# Electroencephalographic Effective Connectivity Analysis of the Neural Networks during Gesture and Speech Production Planning in Young Adults

**DOI:** 10.3390/brainsci13010100

**Published:** 2023-01-04

**Authors:** Yohei Sato, Hiroshi Nishimaru, Jumpei Matsumoto, Tsuyoshi Setogawa, Hisao Nishijo

**Affiliations:** 1 Department of System Emotional Science, Faculty of Medicine, University of Toyama, Toyama 930-0194, Japan; 2 Research Center for Idling Brain Science (RCIBS), University of Toyama, Toyama 930-0194, Japan

**Keywords:** gesture execution, speech production, EEGs, ICs, effective connectivity

## Abstract

Gestures and speech, as linked communicative expressions, form an integrated system. Previous functional magnetic resonance imaging studies have suggested that neural networks for gesture and spoken word production share similar brain regions consisting of fronto-temporo-parietal brain regions. However, information flow within the neural network may dynamically change during the planning of two communicative expressions and also differ between them. To investigate dynamic information flow in the neural network during the planning of gesture and spoken word generation in this study, participants were presented with spatial images and were required to plan the generation of gestures or spoken words to represent the same spatial situations. The evoked potentials in response to spatial images were recorded to analyze the effective connectivity within the neural network. An independent component analysis of the evoked potentials indicated 12 clusters of independent components, the dipoles of which were located in the bilateral fronto-temporo-parietal brain regions and on the medial wall of the frontal and parietal lobes. Comparison of effective connectivity indicated that information flow from the right middle cingulate gyrus (MCG) to the left supplementary motor area (SMA) and from the left SMA to the left precentral area increased during gesture planning compared with that of word planning. Furthermore, information flow from the right MCG to the left superior frontal gyrus also increased during gesture planning compared with that of word planning. These results suggest that information flow to the brain regions for hand praxis is more strongly activated during gesture planning than during word planning.

## 1. Introduction

Gestures, along with speech, play an essential role in face-to-face communication, as gesture and speech form an integrated system [1,2,3]. For example, words representing objects are often accompanied by gestures that represent them (e.g., iconic gestures; [1]). Furthermore, gestures and words representing the same information are produced during time synchronization. Several psychological theories suggest that gestures and speech may initially be produced in the same unit, such as the conceptualizer, to form communicative intention [4,5,6], which might correspond to the “whether decision system” in an early stage of volition to decide whether to make an action [7]. Gestures may also support speech production by facilitating word retrieval [8,9]. A psychological study also reported that specific syllable pronunciations affect specific types of hand-grip performance [10]. These findings suggest that gesture and speech production systems are closely related. Consistently, it has been proposed that language may have evolved from manual and facial gestures [11,12].

Lesion and noninvasive imaging studies have reported neural substrates for gesture and speech production. Lesion studies on apraxia (deficits in skilled movements such as gestures) and aphasia reported that apraxia and aphasia were correlated, suggesting that neural networks for gesture production and word production share the same brain regions [13]. Interestingly, patients with aphasia display deficits in the integration of gestures and speech [14]. Consistently, functional magnetic resonance imaging (fMRI) studies during the planning of gestures and word production suggest that neural networks for gesture production and word production are linked and share similar brain regions, consisting of the fronto-temporo-parietal brain regions [15,16]. Nevertheless, it is also possible that word and gesture production can be controlled independently. Furthermore, some patients display aphasia without apraxia [17,18] or the opposite pattern (i.e., apraxia without aphasia) [19]. These findings suggest that neural networks to control gestures and word production are not identical and flexibly change to produce them.

Recent studies have reported that neural networks connecting different brain regions dynamically change based on the state of subjects and tasks [20,21]. This suggests that some connections in the neural network(s) change dynamically, although the overall neural networks are similar for gesture and word production. Furthermore, recent fMRI studies using diffusion tensor imaging in patients with stroke and schizophrenia having gesture disturbances reported that decreased efficiency of the neural network and disconnection within the neural network were associated with gesture disturbances [22,23]. In addition, patients with schizophrenia display alterations in brain effective connectivity during the detection of mismatched auditory stimuli [24]. Effective connectivity is a measure of directed connectivity that represents the causal influence of one brain region on other brain regions (i.e., information flow from one region to another) [25,26]. Based on these findings, we hypothesized that a common neural network may be involved in both gesture and word production in the initial stage, such as conceptualization, but in a later stage, different neural networks with different information flows would be generated for gesture and word production planning. To investigate differences in dynamic information flow within the neural networks between the planning of gestures and that of word production, electroencephalographs (EEGs) were recorded during the planning, and effective connectivity among different brain regions in the frontal-temporo-parietal network was analyzed.

## 2. Materials and Methods

### 2.1. Participants

Nineteen healthy subjects participated in the current study (30.1 ± 2.2 years, mean age ± standard error of mean; all right-handed; male, n = 13; female, n = 6). The study protocol was carried out in accordance with the Declaration of Helsinki and was reviewed and approved by the Ethics Review Board for Human Research at the University of Toyama (permit no.: R2020052, approved on 4 June 2020). Written informed consent was obtained from all the subjects.

### 2.2. Experimental Procedures

**Figure 1:**
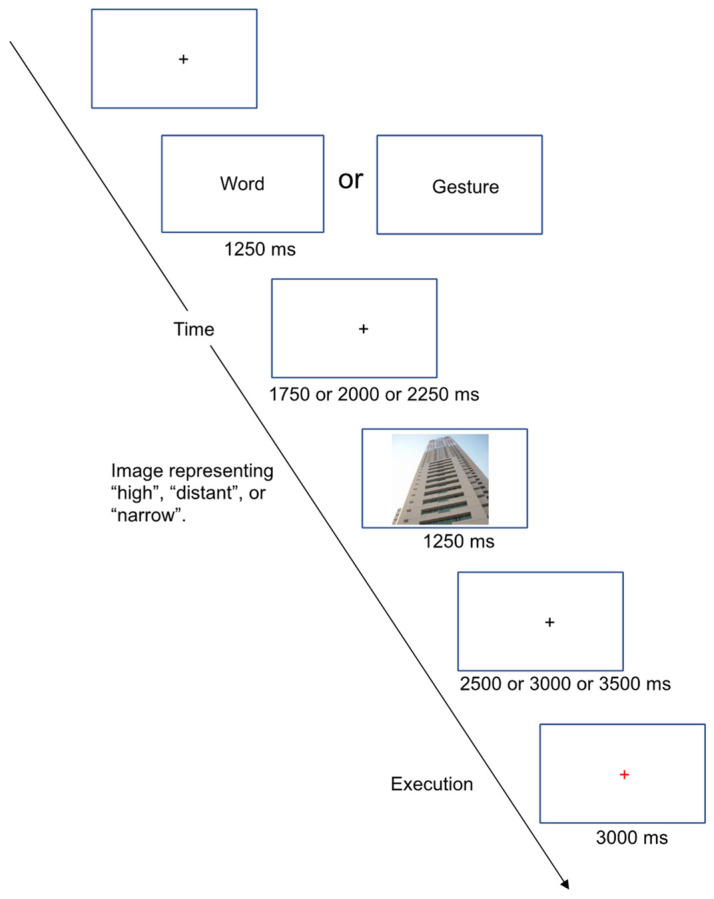
Time course of a gesture and word production (GWP) task.

A subject was seated in a chair in a shielded room and asked to look at a display 70 cm away from the subject on which a black fixation cross was displayed. In a gesture and word production (GWP) task (Figure 1), an instruction word indicating action (gesture or spoken word production: defined as gesture-planning condition and word-planning condition, respectively), which the subject had to perform in the task, initially appeared for 1250 ms. The instruction word was then replaced by the fixation cross. The duration of the black fixation cross was randomly selected from three choices (1750, 2000, or 2250 ms). Then, a spatial image (a picture of a scene) representing “high,” “distant,” or “narrow” was presented for 1250 ms, followed by a black fixation cross, the duration of which was randomly selected among three choices (2500, 3000, or 3500 ms). Finally, the black fixation cross was replaced with a red cross requiring execution of the action (gesture or spoken word production), indicated by the initial instruction word, representing the image. Before the EEG recording, the subjects received instructions about the GWP task and were shown six action samples (three gestures and three words). The subjects were then allowed to perform 18 training trials using their right hand. In this study, the gestures were not associated with the words during the training trials, and the subjects were required to generate only the gestures in the gesture-planning condition during the recording trials.

After the training trials, EEGs (bandpass of 0.018–120 Hz, sampling rate of 500 Hz) were recorded from 64 Ag/AgCl electrodes mounted on the subject’s scalp (EEG-1000, Nihon Kohden, Tokyo, Japan). The impedance of the electrodes was maintained at <30 kΩ. The subjects performed the GWP task in 306 trials, while EEGs were recorded for the subjects. The inter-trial interval was 3 s. The correct performance of the participants was confirmed by audiovisual inspection of the experimenter.

### 2.3. Data Analysis

The stored digitized EEG data were analyzed offline using an open source software (EEGLAB 14.1.2) [27] running on MATLAB 2017b (The MathWorks, Natick, MA, USA), according to previous studies [24,28,29,30]. The EEG data were down-sampled to 100 Hz and high-pass filtered (finite impulse response; Blackman window; cutoff frequency, 0.5 Hz; transition bandwidth, 0.5; filter order, 2816). Recordings from 1 of the 64 channels were found to include large artifacts; therefore, the electrode locations of 63 channels in the MNI coordinate system were imported. Line 60 Hz noises were eliminated using the EEGLAB plugin “CleanLine”. High-amplitude artifacts were removed and reconstructed using the EEGLAB plugin “clean_rawdata(),” including Artifact Subspace Reconstruction (ASR) [31,32,33,34,35,36,37]. For the removal of artifacts with this plugin, the following parameters were used: flat line removal, 5 s; electrode correlation, 0.9; ASR with correction criterion in SD, 15; window rejection with poor quality, 25%. The recordings from the rejected channels were interpolated using the spline interpolation function in EEGLAB. The resultant EEG data were re-referenced to a common average reference. The data epochs for 2 s starting 1 s before the image onset and ending 1 s after the image onset were segmented separately for the gesture and word conditions.

EEG data were decomposed into temporally maximally independent components (ICs) using the adaptive mixture independent component analysis (AMICA) [38]. ICs with topographies, power spectrums, and time courses related to eye blinks, saccades, and muscular artifacts were excluded using the EEGLAB plugin “ICLabel”, and also manually [39,40]. After the exclusion of the ICs not related to brain activity, a total of 460 ICs were selected for all subjects (mean number of brain ICs per condition per person, 12.11 ± 2.33). All ICs were grouped into clusters using the k-means algorithm, and the number of clusters was determined based on the silhouette index [24]. To compute the group-level locations of equivalent current dipoles of the IC clusters (probabilistic dipole density: “probability” in Supplementary Table S1), the estimated locations of the dipoles for the ICs were convolved using a 3D Gaussian kernel [24,28,41] and mapped to the 76 anatomical brain regions based on Automated Anatomical Labeling [24,42].

The mean group-level effective connectivity based on renormalized partial directed coherence (rPDC), frequency domain measure for Granger-causality [43], was computed across ICs with a sliding window using the EEGLAB plugin “groupSift” [24,28,41]. Briefly, the connectivity matrix of IC × IC for individual subjects was estimated with the following parameters: sliding window length, 0.5 s; window step size, 25 ms; frequency range, 2–49 Hz; and number of frequency bins, 30. To compute effective group-level connectivity across the subjects, the IC × IC connectivity matrices for individual subjects were segmented into a 76 × 76 anatomical region matrix (rPDC matrix: rPDC as a weighting factor to modulate pairwise dipole density) with the following parameters: Gaussian smoothing kernel size, 20 mm full width at half maximum; and minimum percentage of subjects with non-zero, 80%. There were 15 out of the 76 anatomical brain regions which showed overlap between the two conditions (gesture vs. word), which constituted 57.0% of total dipole density.

### 2.4. Statistical Analysis

To statistically compare the rPDC matrices between the two conditions (gesture vs. word), uncorrected *t*-tests between the two conditions were performed on each rPDC time-frequency plot at the pixel level and masked at *p* < 0.01. A weak family-wise error rate control was used [24,28]. Briefly, a non-parametric permutation test (n = 10,000) was performed by shuffling the conditions (gesture vs. word planning conditions) of the rPDC matrices, and t-statistics of *t*-tests in the true and surrogate data were compared at *p* < 0.0001 [24].

## 3. Results

### 3.1. ICs during the Planning of Gesture and Word Production

**Figure 2:**
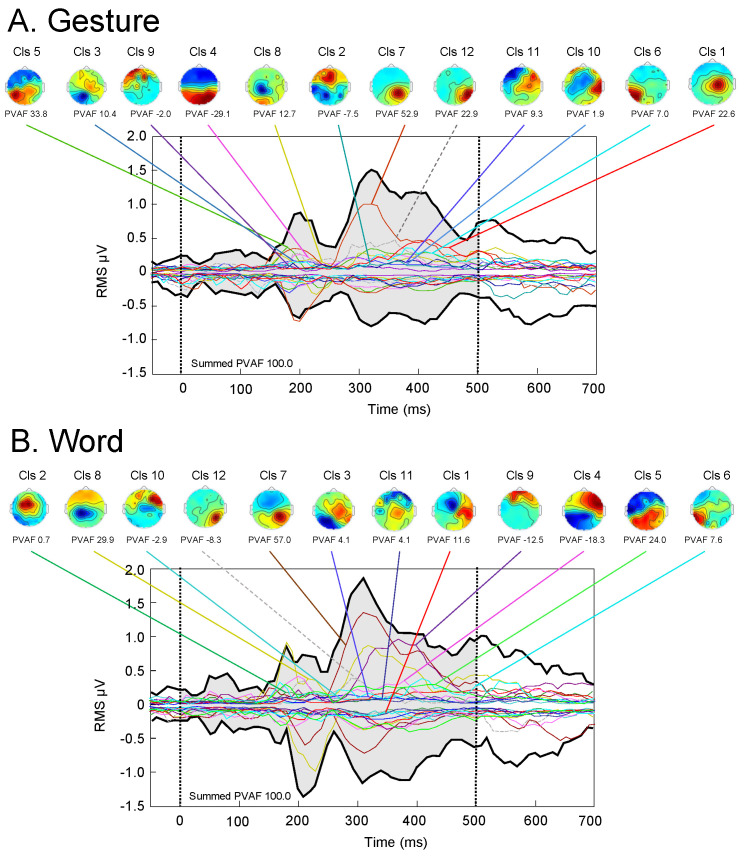
Evoked potentials shown as envelop plots in the gesture (**A**) and word (**B**) planning conditions. Twelve IC clusters were identified. Each colored potential and corresponding topography with each number indicate those of each IC cluster. Zero in the abscissas indicates the onset of images. IC, independent component; Cls, cluster; PVAF, percent variance accounted for.

IC analysis identified 460 ICs, which were grouped into 12 IC clusters based on the Silhouette index (Supplementary Figure S1). Figure 2 shows the root-mean-square (RMS) evoked potentials of the 12 IC clusters in response to the images used for the planning of gesture (Figure 2A) and word (Figure 2B) production. The evoked potentials are shown as envelope plots, in which the maximal and minimal potentials across all electrodes are indicated in each time frame. Supplementary Figure S2 shows the individual RMS evoked potentials of the 12 IC clusters in the gesture and word planning conditions (detailed information on how to represent ICs is provided on https://sccn.ucsd.edu/wiki/Makoto’s_preprocessing_pipeline (accessed on 30 December 2022).

### 3.2. Neural Networks during the Planning of Gesture and Word Production

**Figure 3:**
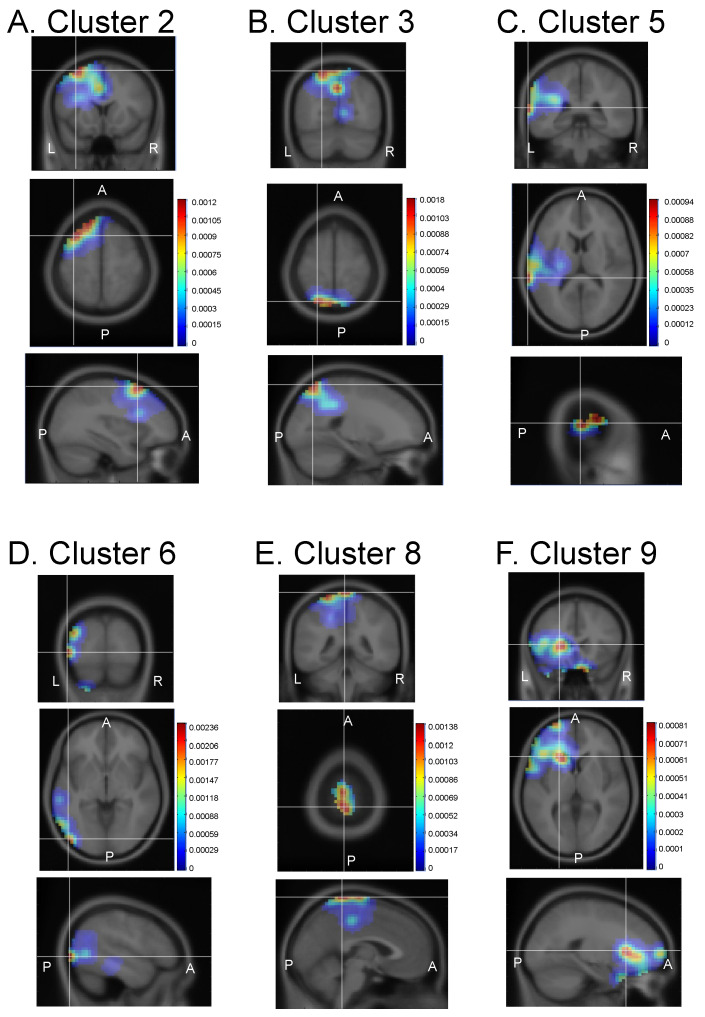
Probabilistic dipole density of the six IC clusters identified in the left hemisphere. (**A**–**F**) Dipole density in each IC cluster. In each IC cluster, the top, middle, and bottom panels indicate coronal, horizontal, and sagittal planes, respectively. L, left; R, right; A, anterior; P, posterior. Color calibration bars indicate probabilistic dipole density. IC, independent component.

**Figure 4:**
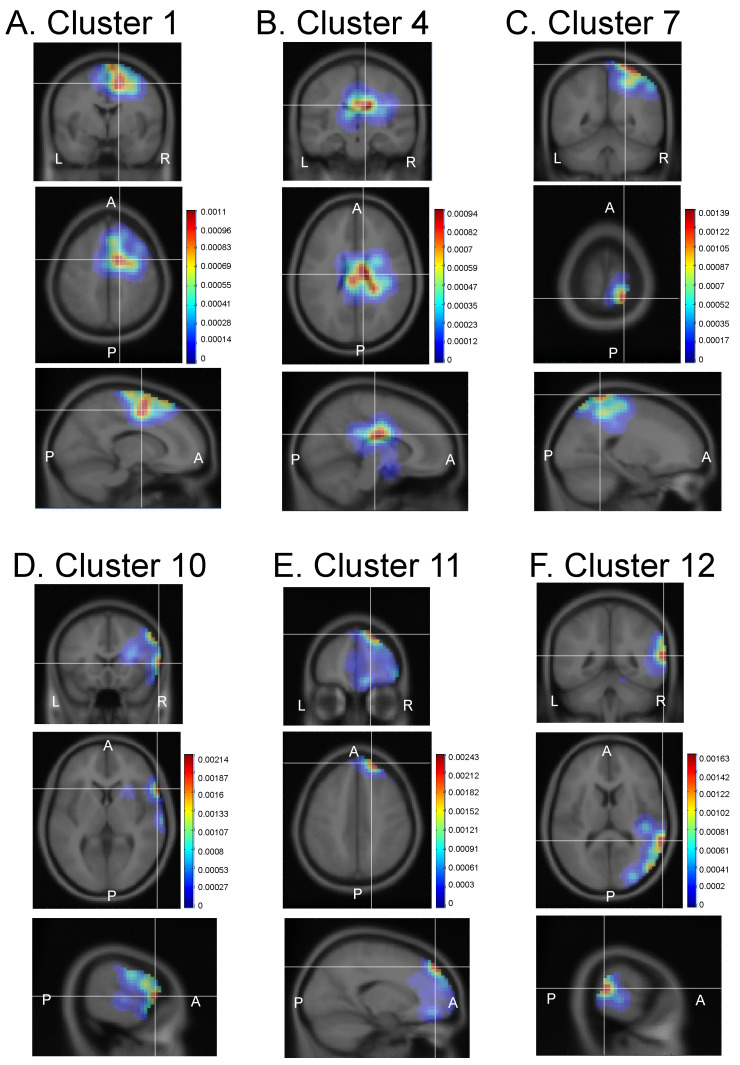
Probabilistic dipole density of the six IC clusters identified in the right hemisphere. (**A**–**F**) Dipole density in each IC cluster. In each IC cluster, the top, middle, and bottom panels indicate coronal, horizontal, and sagittal planes, respectively. L, left; R, right; A, anterior; P, posterior. Color calibration bars indicate probabilistic dipole density. IC, independent component.

The probabilistic locations of the 12 IC clusters are shown in Figure 3 and Figure 4, and their probabilistic plots (dipole density) in the brain regions based on the brain atlas with Talairach coordinates [44] are shown in Supplementary Table S1. Six clusters were identified in the left hemisphere (Figure 3). Dipoles of IC cluster 2 were densely located in the left middle and superior frontal gyri (Figure 3A). Dipoles of IC cluster 3 were densely located around the left parietal lobe, including the inferior parietal lobule, precuneus, and superior parietal lobule (Figure 3B). Dipoles of IC cluster 5 were densely located around the left temporal lobe, including the superior and middle temporal gyri (Figure 3C). Dipoles of IC cluster 6 were densely located around the left occipitotemporal regions, including the left inferior and superior temporal gyri and the left middle occipital gyrus (Figure 3D). Dipoles of IC cluster 8 were densely located around the medial regions of the left parietal lobe, including the left paracentral lobule and precuneus (Figure 3E). Dipoles of IC cluster 9 were densely located around the left frontal lobe, including the anterior cingulate gyrus, inferior frontal gyrus, and insula (Figure 3F).

Six clusters were identified in the right hemisphere (Figure 4). Dipoles of IC cluster 1 were densely located around the posteromedial regions of the right frontal lobe, including the paracentral lobule, middle cingulate gyrus, and medial frontal gyrus (Figure 4A). Dipoles of IC cluster 4 were densely located around the medial regions of the right parieto-frontal lobe, including the right middle and posterior cingulate gyri (Figure 4B). Dipoles of IC cluster 7 were densely located around the medial regions of the right parietal lobe, including the right paracentral lobule, postcentral gyrus, precuneus, and superior parietal lobule (Figure 4C). Dipoles of IC cluster 10 were densely located around the right frontal lobe, including the right inferior frontal gyrus, insula, and precentral gyrus (Figure 4D). Dipoles of IC cluster 11 were densely located around the anterior regions of the right frontal cortex, including the right medial frontal gyrus, middle cingulate gyrus, and superior frontal gyrus (Figure 4E). Dipoles of IC cluster 12 were densely located around the right parieto-occipital regions, including the right middle, superior, and inferior temporal gyri, and the inferior parietal lobule (Figure 4F). Thus, the results indicated that neural network(s) consisting of the fronto-temporo-parietal brain regions were active during the planning of gesture and word production. The locations of the 12 IC clusters in the gesture- (A) and word- (B) planning conditions are shown together in the same brain model in Supplementary Figure S3.

### 3.3. Effective Connectivity during the Planning of Gesture and Word Production

**Figure 5:**
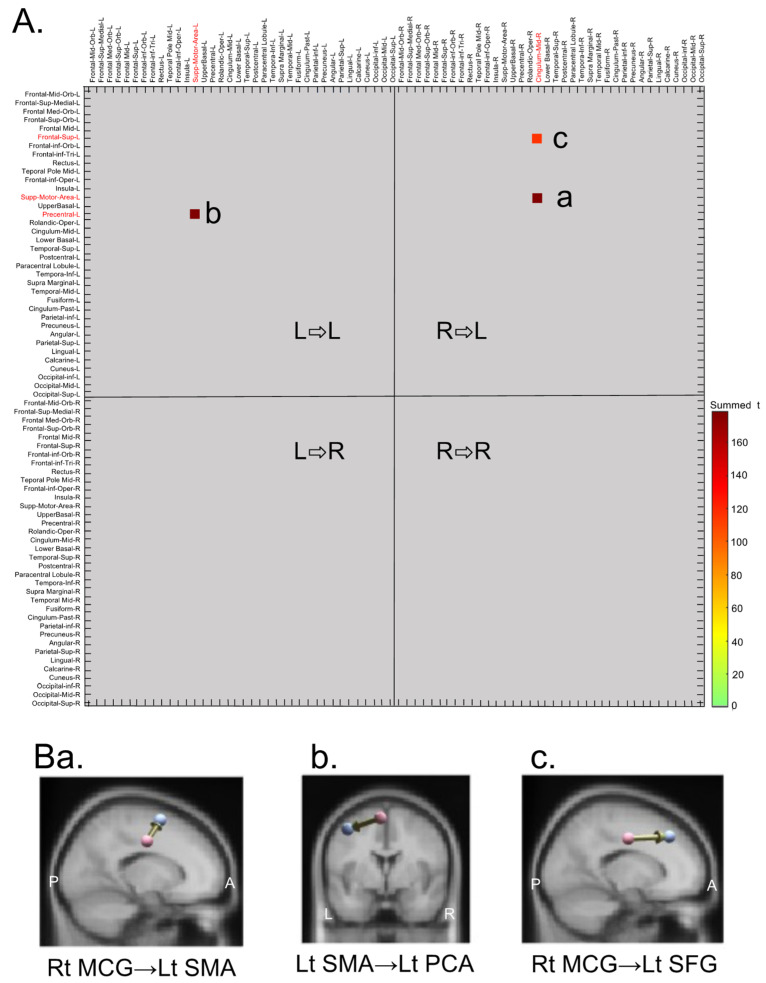
Summary of the effective connectivity differences between the gesture and word planning conditions shown as a matrix plot (**A**) and directed arrows (**B**). (**A**) Each colored square indicates a significant difference between the two conditions (corrected, *p* < 0.0001). L ⇨ L, connectivity from the left to the left hemisphere; R ⇨ L, connectivity from the right to the left hemisphere; L ⇨ R, connectivity from the left to the right hemisphere; R ⇨ R, connectivity from the right to the right hemisphere. Cingulum-Mid-R, right middle cingulate gyrus (MCG); Supp-Motor-Area-L, left supplementary motor area (SMA); Precentral-L, left precentral area (PCA); Frontal-Sup-L, left superior frontal gyrus (SFG). (**B**) Three connectivities with significant differences between the two conditions. Arrows in (**a**–**c**) indicate directed connectivity shown in a, b, and c in (**A**), respectively. Rt, right; Lt, left; L, left; R, right; A, anterior; P, posterior.

Figure 5A shows a connectivity matrix with a significant difference at *p* < 0.0001 (corrected: [24]) between the two planning conditions. This comparison indicated significant differences in the three connections: the connection from the right middle cingulate gyrus (MCG) to the left supplementary motor area (SMA) (Aa), from the left SMA to the left precentral area (PCA) including the primary motor cortex (Ab), and from the right MCG to the left superior frontal gyrus (SFG) (Ac). These three connections are shown in Figure 5B. Significant connectivity differences between the two conditions were initially observed in connectivity from the right MCG to the left SMA, followed by connectivity from the left SMA to the left PCA, and from the right MCG to the left SFG (see below for details). 

**Figure 6:**
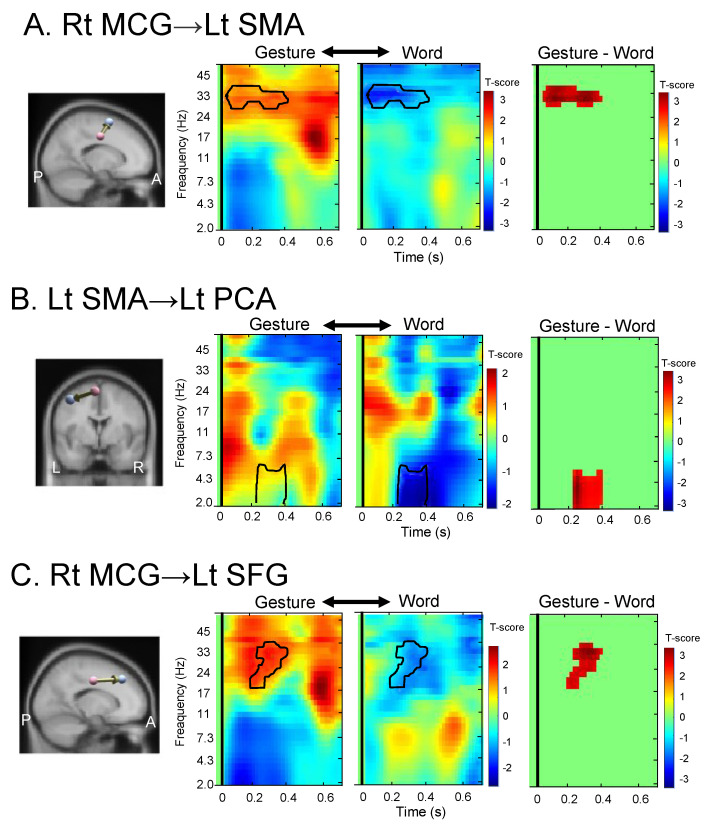
Time-frequency plots of renormalized partial directed coherence (rPDC) in the three effective connectivity with significant differences between the gesture and word planning conditions, as shown in Figure 5. (**A**–**C**) rPDC plots in the effective connectivity from the right MCG to the left SMA (**A**), from the left SMA to the left PCA (**B**), and from the right MCG to the left SFG (**C**). Areas surrounded by solid lines indicate significant differences in rPDCs between the two planning conditions. Zero in the abscissas indicates the onset of images. Rt, right; Lt, left; L, left; R, right; A, anterior; P, posterior.

Figure 6 shows the time-frequency plots of the rPDC for these connectivities. This analysis was performed without any prior hypothesis regarding connectivity changes in a specific time-frequency range. In the connectivity from the right MCG to the left SMA (Figure 6A), the connectivity of around 33 Hz in the low gamma band increased in the gesture-planning condition immediately after the image onset, while this connectivity decreased in the word-planning condition. In the connectivity from the left SMA to the left PCA (Figure 6B), the connectivity of around 2–4 Hz in the delta band increased in the gesture-planning condition around 0.2 s after the image onset, while this connectivity was decreased in the word-planning condition. Thus, the connectivity in the right MCG–left SMA–left PCA pathway was significantly increased in the gesture-planning condition compared with that in the word planning condition. In the connectivity from the right MCG to the left SFG (Figure 6C), the connectivity of around 17–33 Hz in the beta and low gamma bands increased in the gesture-planning condition 0.2 s after the image onset, while this connectivity was decreased in the word-planning condition. However, no connectivity increased in the word-planning condition compared with that in the gesture-planning condition.

## 4. Discussion

### 4.1. Neural Networks during the Planning of Gesture and Word Production

Consistent with previous fMRI studies [15,16], 12 clusters of ICs were estimated in the neural network(s) consisting of the fronto-temporo-parietal brain regions during planning of gesture and word production. The ICs were estimated in the left and right superior parietal lobules (SPL). Previous studies have reported that the left or bilateral SPL is active during hand movements for signing [45] and is involved in linguistic working memory [46], control of learned motor movements [47], and visual-spatial attention [48,49]. Thus, the SPL might be active under both planning conditions in the present study.

ICs were also estimated in the bilateral precentral gyrus and left insula. A previous study reported that the bilateral precentral gyrus and left insula were active during three tasks, including silent word production, non-speech mouth movements, and finger movements [50]. Furthermore, ICs were estimated in the inferior frontal gyrus, including the Broca’s area, inferior parietal lobule, middle temporal gyrus, anterior cingulate gyrus, and SMA. Previous fMRI and positron emission tomography studies have consistently reported that gesture production and reaching movements activate distributed brain regions, including these brain regions [51,52,53,54]. In addition, lesion and fMRI studies on word or speech production and their planning have also identified similar brain regions [55,56,57]. Thus, most brain regions identified in the present study have been reported to be associated with the planning or execution of both gesture and word production.

### 4.2. Differential Activity between the Planning of Gesture and Word Production

The present study indicated that information flow from the right MCG to the left SMA and from the left SMA to the left PCA was significantly increased in the gesture-planning condition compared with that in the word-planning condition. Some evidence indicates the involvement of these neural circuits in hand control. A previous study reported that the MCG ipsilateral to the hand is active during gesture planning [58]. Patients with schizophrenia having deficits in gesture production display reduced gray matter volume in the right anterior cingulate gyrus and right MCG [59]. The MCG includes the cingulate motor area (CMA) [60]. Monkey CMA neurons are active during unilateral contralateral hand movements as well as unilateral ipsilateral hand movements [61]. Anatomical studies have reported that the CMA projects not only to the ipsilateral SMA but also to the contralateral SMA in monkeys [62,63], while an fMRI study reported that directed functional connectivity from the MCG to the SMA increased during fine finger movements in humans [64]. Furthermore, the CMA was active with concomitant activation of the lateral prefrontal cortex, suggesting that the CMA may transmit cognitive or motor commands from the prefrontal cortex to motor-related areas in humans [65]. The MCG has been implicated in selection for action in various tasks [60]. The present results, along with these findings, suggest that the right MCG might be involved in the selection of hand actions based on information from the lateral prefrontal cortex, and that this selection might be sent to the left SMA. 

Furthermore, this information might be sent from the left SMA to the left PCA, including the motor cortex, in delta oscillation of approximately 2–4 Hz. Movement-related delta oscillations have been reported in the contralateral motor cortex and midline brain areas [66], which may reflect decisions regarding hand selection [67] and may be involved in the organization of cortico-basal ganglia networks [68]. These findings suggest that the information flow in delta oscillations from the left SMA to the left PCA includes critical motor planning information. It is noted that all participants were right-handed in the present study. Therefore, the left-lateralized information flow observed in the gesture-planning condition could be ascribed to right-handedness. Further studies with left-handed participants are required to determine whether the left-lateralized information flow observed in the gesture-planning condition is ascribed to right-handedness.

The present study also indicated that information flow from the right MCG to the left SFG was significantly increased in the gesture-planning condition compared with the word-planning condition. It has been reported that the left SFG is morphologically and functionally altered in schizophrenia and developmental coordination disorder (DCD) [69,70,71], while schizophrenia and DCD show deficits in hand gestures and those in age-appropriate motor skills, respectively [72,73]. Furthermore, the left SFG has been implicated in working memory, motor imagery, and the control of complex hand movements [74,75,76]. These findings suggest that the SFG may be involved in the planning and imagery of gestures based on information from the MCG.

In contrast, word-planning-dominant activation was not identified in the precentral gyrus in the present study. Previous studies on human electrocorticographic and magnetoencephalographic recording reported a putative role of gamma oscillations in both gesture and speech processing [77,78], and that both oral movements for spoken word production and hand movements for sign language increased gamma power in the same ventral part of the precentral area, while hand movements for sign language increased gamma power more strongly in the dorsal part of the precentral area than oral movements for spoken word production [79]. Furthermore, some brain regions (e.g., SMA/post-central regions) are reported to be involved in not only speech but also gesture processing [80,81]. These differences in activation patterns might lead to gesture-planning-condition-dominant (but not word-planning-dominant) information flow to the left precentral gyrus.

## 5. Conclusions

Previous fMRI studies have suggested that neural networks for gesture production and word production are linked and share similar brain regions, consisting of the fronto-temporo-parietal brain regions. Recent studies have suggested that neural information flow and networks are dynamically altered and that dynamically changing information flow within neural networks may differ between the two communicative expressions. In the present study, to investigate dynamic information flow in neural networks during the planning of gesture and word generation, evoked potentials in response to spatial images were recorded in the GWP task. The subjects were required to plan gestures or generate words to represent the spatial situations of the images in the task. The results indicated that evoked potentials consisted of 12 clusters of ICs, the dipoles of which were located in the bilateral fronto-temporo-parietal brain regions as well as the medial wall of the frontal and parietal lobes. Effective connectivity analysis of these ICs indicated that gesture planning, compared with that of word production, increased information flow from the right MCG to the left SMA and from the left SMA to the left PCA. Furthermore, gesture planning increased the information flow from the right MCG to the left SFG compared with that in the planning of word production. These results suggest that the neural circuits for hand praxis are more strongly activated during the planning of gesture generation than during word generation. These differences in dynamic information flow within similar neural circuits may reflect the planning of different motor actions (gestures or word production) that represent the same meaning.

## Data Availability

The data presented in this study are available in Supplementary Materials. The other data presented in this study are available on request from the corresponding author.

## Supplementary Materials

The following supporting information can be downloaded at: https://www.mdpi.com/article/10.3390/brainsci13010100/s1, Figure S1: Relationships between number of IC clusters and Silhouette index on k-means clustering; Figure S2: RMS evoked potentials of each IC cluster in the gesture and word planning conditions (A–L); Figure S3: Locations of the 12 IC clusters in the gesture- (A) and word- (B) planning conditions; Table S1: Distributions of probabilistic dipole density (probability) in the 12 IC clusters.
