# Supplementary material for: Electroencephalographic Effective Connectivity Analysis of the Neural Networks during Gesture and Speech Production Planning in Young Adults
Source: Brain Sci. 2023 Jan 4;13(1):100. doi: 10.3390/brainsci13010100 (PMC9856316; doi:10.3390/brainsci13010100)
Supplement: Supplementary file 1 [file brainsci-13-00100-s001.zip › brainsci-2085879-supplementary.pdf]

Electroencephalographic effective connectivity analysis of the neural networks during gesture and speech production planning in young adults

Sato et al.

Supplementary information

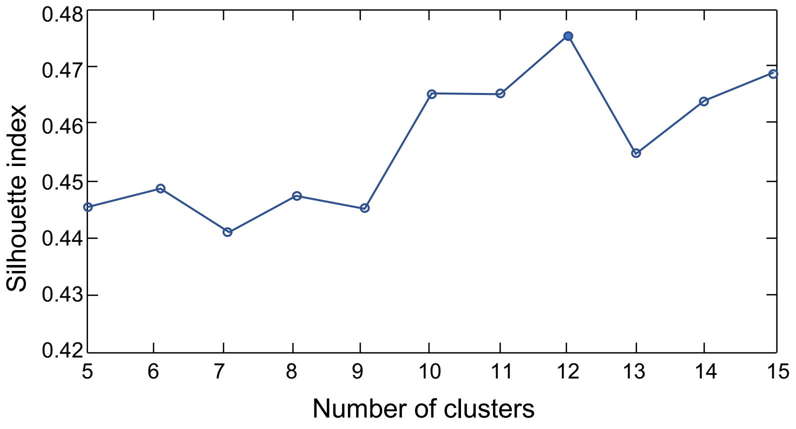

Supplementary Figure S1. Relationships between number of IC clusters and Silhouette index on k-means clustering.

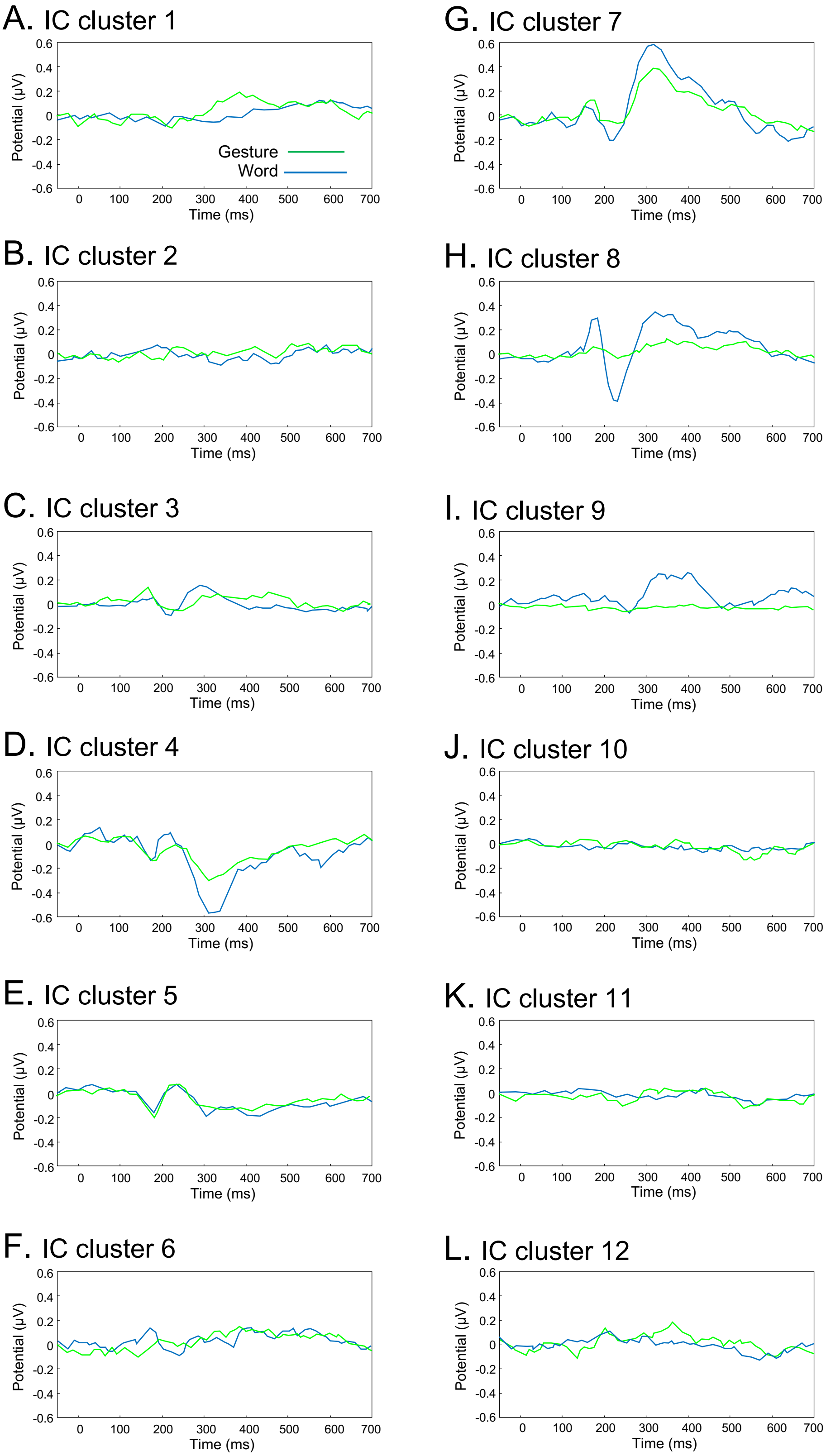

Supplementary Figure S2. RMS evoked potentials of each IC cluster in the gesture and word planning conditions (A-L). Zero in the abscissas indicate onset of the images. Green and blue lines indicate the gesture and word planning conditions, respectively.

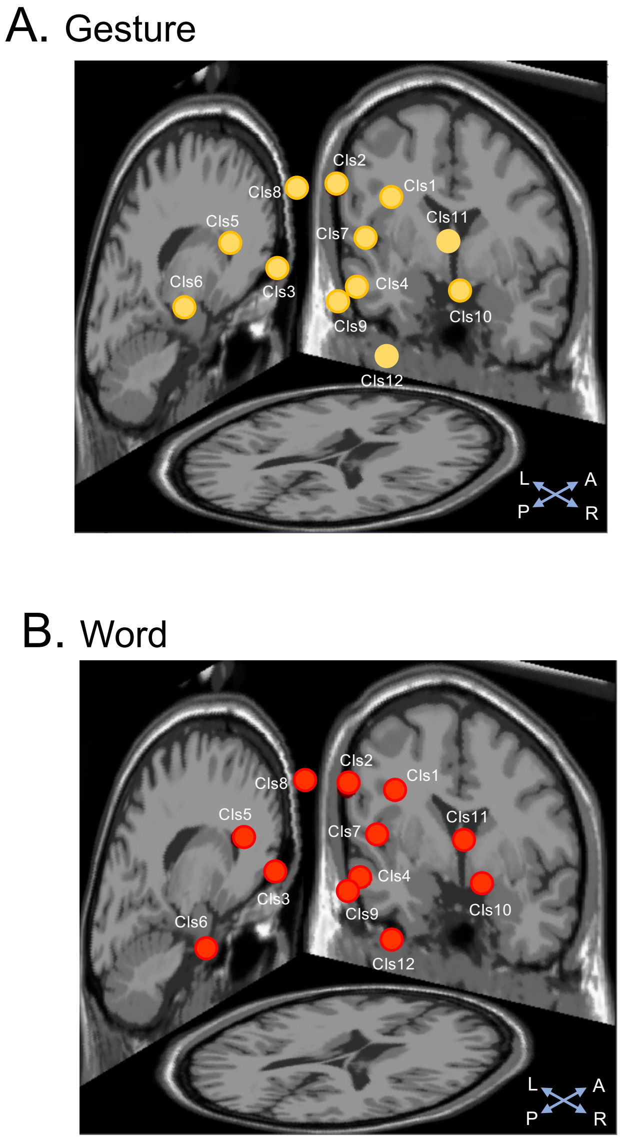

Supplementary Figure S3. Locations of the 12 IC clusters in the gesture- (A) and word- (B) planning conditions. Each cluster was plotted according to the mean MNI coordinates of the individual ICs included in each IC cluster. Cls, IC cluster; A, anterior; P, posterior; L, left; R, right.

Supplementary Table S1. Distributions of probabilistic dipole density (probability) in the 12 IC clusters.

	Brain regions	Probability
Cluster 1	Rt paracentral lobule	0.273
	Rt middle cingulate gyrus	0.212
	Rt medial frontal gyrus	0.152
	Rt middle frontal gyrus	0.091
	Rt superior frontal gyrus	0.061
	Rt precentral gyrus	0.03
Cluster 2	Lt middle frontal gyrus	0.211
	Lt superior frontal gyrus	0.211
	Lt precentral gyrus	0.158
	Lt middle cingulate gyrus	0.053
	Lt medial frontal gyrus	0.053
Cluster 3	Lt inferior parietal lobule	0.263
	Lt precunes	0.263
	Lt superior parietal lobule	0.211
Cluster 4	Rt middle cingulate gyrus	0.229
	Rt posterior cingulate gyrus	0.125
Cluster 5	Lt superior temporal gyrus	0.256
	Lt middle temporal gyrus	0.191
	Lt inferior parietal lobule	0.106
	Lt postcentral gyrus	0.106
	Lt supramarginal gyrus	0.085
	Lt insula	0.064
	Lt transverse temporal gyrus	0.043
Cluster 6	Lt inferior temporal gyrus	0.177
	Lt middle temporal gyrus	0.177
	Lt middle occipital gyrus	0.152
	Lt fusiform gyrus	0.114
	Lt lingual gyrus	0.063
	Lt cuneus	0.038

	Brain regions	Probability
Cluster 7	Rt paracentral lobule	0.188
	Rt postcentral gyrus	0.156
	Rt precunes	0.156
	Rt superior parietal lobule	0.156
	Rt inferior parietal lobule	0.063
Cluster 8	Lt paracentral lobule	0.37
	Lt precunes	0.13
	Lt superior parietal lobule	0.065
	Lt precentral gyrus	0.022
Cluster 9	Lt anterior cingulate gyrus	0.138
	Lt inferior frontal gyrus	0.138
	Lt. insula	0.087
	Lt medial frontal gyrus	0.087
	Lt middle frontal gyrus	0.075
	Lt middle cingulate gyrus	0.05
	Lt precentral gyrus	0.05
Cluster 10	Rt inferior frontal gyrus	0.271
	Rt insula	0.188
	Rt precentral gyrus	0.146
	Rt superior temporal gyrus	0.104
	Rt middle frontal gyrus	0.063
Cluster 11	Rt middle cingulate gyrus	0.256
	Rt medial frontal gyrus	0.256
	Rt superior frontal gyrus	0.186
	Rt middle frontal gyrus	0.093
Cluster 12	Rt middle temporal gyrus	0.174
	Rt superior temporal gyrus	0.159
	Rt inferior temporal gyrus	0.159
	Rt inferior parietal lobule	0.087
	Rt insula	0.087
	Rt supramarginal gyrus	0.087
	Rt middle occipital gyrus	0.058
	Rt postcentral gyrus	0.043
	Rt transvers temporal gyrus	0.029

Lt, left; Rt, right.
